# PEGylating a bacteriophage endolysin inhibits its bactericidal activity

**DOI:** 10.1186/2191-0855-1-29

**Published:** 2011-10-07

**Authors:** Gregory Resch, Philippe Moreillon, Vincent A Fischetti

**Affiliations:** 1 Laboratory of Bacterial Pathogenesis and Immunology, The Rockefeller University, 1230 York Avenue, New York, NY 10021, USA; 2 Department of Fundamental Microbiology, University of Lausanne, UNIL-Sorge, Biophore Building, CH-1015 Lausanne, Switzerland

**Keywords:** Bacteriophage, *S. pneumoniae*, Cpl-1, PEGylation, Endolysin, Enzybiotic

## Abstract

Bacteriophage endolysins (lysins) bind to a cell wall substrate and cleave peptidoglycan, resulting in hypotonic lysis of the phage-infected bacteria. When purified lysins are added externally to Gram-positive bacteria they mediate rapid death by the same mechanism. For this reason, novel therapeutic strategies have been developed using such enzybiotics. However, like other proteins introduced into mammalian organisms, they are quickly cleared from systemic circulation. PEGylation has been used successfully to increase the *in vivo *half-life of many biological molecules and was therefore applied to Cpl-1, a lysin specific for *S. pneumoniae*. Cysteine-specific PEGylation with either PEG 10K or 40K was achieved on Cpl-1 mutants, each containing an additional cysteine residue at different locations To the best of our knowledge, this is the first report of the PEGylation of bacteriophage lysin. Compared to the native enzyme, none of the PEGylated conjugates retained significant *in vitro *anti-pneumococcal lytic activity that would have justified further *in vivo *studies. Since the anti-microbial activity of the mutant enzymes used in this study was not affected by the introduction of the cysteine residue, our results implied that the presence of the PEG molecule was responsible for the inhibition. As most endolysins exhibit a similar modular structure, we believe that our work emphasizes the inability to improve the *in vivo *half-life of this class of enzybiotics using a cysteine-specific PEGylation strategy.

## Introduction

*Streptococcus pneumoniae *is the first cause of otitis media and a common cause of sinusitis, community-acquired pneumonia, bacteremia, and meningitis (Jacobs, 2004). Antibiotic misuse and overuse has progressively selected for resistance against major drug classes, and treatment failures are widely reported (Fuller and Low, 2005; Klugman, 2002). This justifies the search for new drugs with different mechanisms of action. The bacteriolytic action of bacteriophage lysins enables the release of phage progeny from the bacterial sacculus. Purified pneumococcal phage lysin Cpl-1 has been used to successfully treat pneumococcal sepsis, endocarditis, meningitis, and pneumonia in rodent models (Entenza et al., 2005; Grandgirard et al., 2008; Loeffler et al., 2003). However, due to its short circulating half-life (~20.5 minutes) (Loeffler et al., 2003), optimal efficacy requires repeated injections or continuous infusion (Entenza et al., 2005). We recently showed that pre-dimerization of Cpl-1, which doubles the molecular weight of the enzyme, decreased its plasma clearance by a factor of ten (Resch et al., 2011). PEGylation (Veronese and Pasut, 2005) was shown to extend even more so the serum half-life of interferon-α2b from minutes to hours (Ramon et al., 2005) and of lysostaphin from 1 to 24 h (Walsh et al., 2003). Here we mono-PEGylated (Gaberc-Porekar et al., 2008; Walsh et al., 2003) Cpl-1 at various cysteine residues and determined the anti-pneumococcal activity of the resulting conjugates.

## Materials and methods

### Reagents

Plasmid mini-prep kits were bought from Qiagen (Valencia, CA, USA). The QuickChange II Site-Directed Mutagenesis Kit was purchased from Stratagene (Cedar Creek, TX, USA). Mutagenic primers were obtained from Fischer Biotechnology (Pittsburgh, PA, USA) and DNA sequencing reactions were performed by Genewiz (South Plain, NJ, USA). DEAE-Sepharose, HiLoad 16/60 SuperdexTM 200 prep grade column, and PD-10 desalting columns were obtained from GE Healthcare Bio-Sciences Corp. (Piscataway, NJ, USA). Amicon Ultra centrifugal units Ultracel 30K were from Millipore (Carrigtwohill, Co. Cork, Ireland). Chemically competent *Escherichia coli *(*E. coli*) Max Efficiency DH5α cells and NuPAGE 4-12% Bis-Tris Gels were from Invitrogen (Carlsbad, CA, USA). Poly-ethylene glycol maleimide MW 10 kDa (PEG 10) and Y-shape poly-ethylene glycol maleimide MW 40 kDa (PEG 40) were purchased from Jenkem Technology (Allen, TX, USA). All other chemicals were from Sigma-Aldrich (Saint Louis, MO. USA).

### Choosing PEGylation sites

In the present study, seven mutants previously described elsewhere as showing comparable antimicrobial activity to parent Cpl-1 were included (Resch et al., 2011). The mutants are as follows: Cpl-1^C45S;Q85C ^Cpl-1^C45S;D194C ^Cpl-1^C45S;N214C ^Cpl-1^C45S;G216C ^Cpl-1^C45S;D256C ^Cpl-1^C45S;S269C ^Cpl-1^C45S;D324C ^(Table 1). A previous study on lysostaphin PEGylation suggesting that future studies should focus on mono-PEGylation in order to prevent total inhibition of enzyme activity (Walsh et al., 2003), led us to choose to neo-introduce a single exposed cysteine in our Cpl-1 mutants. The nucleotide sequence of Cpl-1 can be access from the Genbank database with accession number NC_001825.

**Table1:** List of mutagenic primers used in site-directed mutagenesis experiments

Cpl-1 mutant	Forward mutagenic primer	Reverse mutagenic primer
Cpl-1^C45S^	5'-CGA CCT ATT TAA ACC CTA GCT TGT CTG CTC AAG TGG AGC AGT CAA ACC C-3'	5'-GGG TTT GAC TGC TCC ACT TGA GCA GAC AAG CTA GGG TTT AAA TAG GTC G-3'

Cpl1^C45S;Q85C^	5'-GTT TTT CCT TGA CAA CGT GCC TAT GTGCGT TAA ATA CCT TGT ATT GGA CTA CG-3'	5'-CGT AGT CCA ATA CAA GGT ATT TAA CGCACA TAG GCA CGT TGT CAA GGA AAA AC-3'

Cpl1^C45S;D194C^	5'-GTT AGA CGA TGA AGA AGA CTG CAA GCC AAA GAC CGC TGG A-3'	5'-TCC AGC GGT CTT TGG CTT GCA GTC TTC TTC ATC GTC TAA C-3'

Cpl1^C45S;N214C^	5'-GGG TGG TGG TTC AGA CGA TGC AAT GGC AGT TTC CCT TA-3'	5'-TAA GGG AAA CTG CCA TTG CAT CGT CTG AAC CAC CAC CC-3'

Cpl-1^C45S;G216C^	5'-GTG GTG GTT CAG ACG AAA CAA TTG CAG TTT CCC TT-3'	5'-AAG GGA AAC TGCAAT TGT TTC GTC TGA ACC ACC AC-3'

Cpl-1^C45S;D256C^	5'-AAA TGG TAC TAC CTC AAG TGC AAC GGC GCA ATG GCG AC-3'	5'-GTC GCC ATT GCG CCG TTG CAC TTG AGG TAG TAC CAT TT-3'

Cpl-1^C45S;S269C^	5'-GTT GGG TGC TAG TCG GGT GCG AGT GGT ATT ATA TGG AC-3'	5'-GTC CAT ATA ATA CCA CTC GCA CCC GAC TAG CAC CCA AC-3'

Cpl-1^C45S;D324C^	5'-ACA CAA ACG GAG AGC TTG CATGCA ATC CAA GTT TCA CGA AAG-3'	5'-CTT TCG TGA AAC TTG GAT TGCATG CAA GCT CTC CGT TTG TGT-3'

Mutated positions are underlined.

### Construction of plasmids carrying mutated Cpl-1 genes

Plasmids carrying the genes encoding for the Cpl-1 mutants included in this study were constructed as described elsewhere (Resch et al., 2011). Briefly, the plasmid encoding Cpl-1^C45S ^was constructed using the QuickChange II Site-Directed Mutagenesis Kit with appropriate primers (Table 1) in order to introduce the desired mutation in the Cpl-1 gene originally carried on the pJML6 plasmid (Loeffler et al., 2003), following the manufacturer instructions. The plasmids encoding the mutant Cpl-1 proteins were further generated by the same approach (Table 1 for the list of primers) using the plasmid carrying the Cpl-1^C45S ^gene as template. Plasmids containing the mutated genes were further transformed in *E. coli *DH5α following the manufacturer protocol. The presence of the mutations was confirmed by DNA sequencing.

### Production and purification of Cpl-1 mutants

The production and purification of all proteins followed a protocol that has already been described for Cpl-1 (Loeffler and Fischetti, 2003) and Cpl-1 mutants (Resch et al., 2011). Briefly, *E. coli *DH5α cells were grown in Luria-Broth (LB) for 16 h aerobically at 37°C with agitation at 250 rpm. The cultures were diluted 10X (vol/vol) and allowed to grow for an additional 5 h in the same conditions. Protein expression was induced by the addition of 2% (w/v) lactose to the cultures. 16 h later, cells were pelleted, resuspended in phosphate buffer 50 mM, pH 7.4 (enzyme buffer), and sonicated on ice (three cycles of 30 sec at 70% power, Sonoplus, Bandelin Electronics, Berlin, Germany). Cell debris was pelleted by centrifugation (1 h at 4°C and 15,000 rpm) and supernatants were treated with 20 units (20 U) of DNAse I for 16 h at 4°C. 0.45 μm filtered supernatants were applied to a DEAE-Sepharose fast flow column previously equilibrated with enzyme buffer. Following a wash step with enzyme buffer containing 1 M NaCl, the enzymes were eluted with enzyme buffer containing 10% (w/v) choline. After extensive dialysis (cutoff 30,000 kDa) against enzyme buffer, the purified enzymes were concentrated using Ultracel 30K centrifugal filters and stored at -20°C.

### PEGylation of Cpl-1 mutants

Purified mutant enzymes were reduced for 30 min at room temperature (RT) in enzyme buffer containing 10 mM dithiotreitol (DTT), and desalted on PD-10 columns previously equilibrated with enzyme buffer. Protein concentrations were adjusted to 1 mg/ml and either PEG maleimide MW 10,000 kDa (PEG 10K) or Y-shaped PEG Maleimide MW 40,000 kDa (PEG 40K) was added (1/25 and 1/10 mol protein/mol PEG for PEG 10K and 40K, respectively). After a 15 min. incubation period at RT with constant gentle agitation, the excess of unbound PEG was removed by applying the mixtures to a DEAE-Sepharose column previously equilibrated with enzyme buffer. PEGylated conjugates and residual fractions of non-PEGylated enzymes were eluted with enzyme buffer containing 10% (w/v) choline, and then purified by gel filtration on a HiLoad 16/60 Superdex™ 200 prep grade column pre-equilibrated in enzyme buffer. Fractions containing the purified PEGylated enzymes were pooled, concentrated using Ultracel 30K centrifugal filters and stored at -20°C until further use.

### *In vitro *killing assay

The killing assay was performed using *S. pneumoniae *strain DCC1490 (serotype 14) obtained from A. Tomasz and has been described elsewhere (Loeffler and Fischetti, 2003; Loeffler et al., 2001). Briefly, DCC1490 was grown to log-phase in aerobic conditions without agitation (OD_595 nm _of 0.3) in brain heart infusion (BHI) at 37°C. After centrifugation and re-suspension of DCC1490 in enzyme buffer at a concentration of 10^9 ^cfu/ml, serial dilutions of enzymes were added to the cells. Reaction kinetics were obtained by measuring the decrease of the OD_595 nm _at 37°C over a period of 28 min. in a EL808 microplates reader (Biotek Instruments Gmbh, Luzern, Switzerland).

## Results

**Figure 1:**
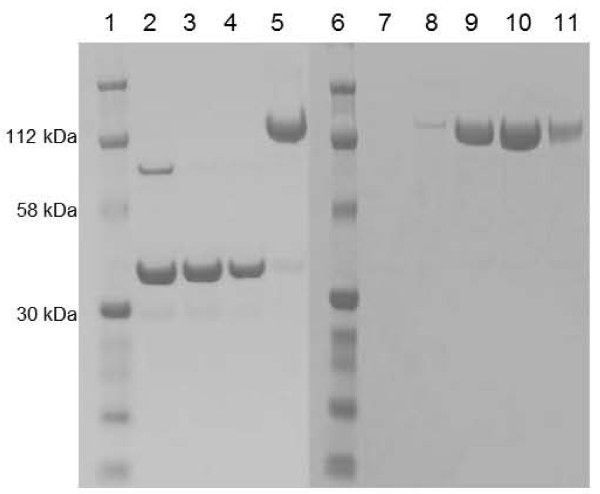
**Non-reducing SDS-PAGE of Cpl-1^C45S;D194C ^PEGylated with PEG 40K**. Protein ladder (lanes 1 and 6); non-reduced Cpl-1^C45S;D194C ^(lane 2); Cpl-1^C45S;D194C ^reduced with 10 mM DTT before and after desalting on a PD-10 column (lane 3 and 4, respectively); Cpl-1^C45S;D194C ^PEGylated with PEG 40K and purified on a DEAE-sepharose column (lane 5); further purification of Cpl-1^C45S;D194C ^PEGylated with PEG 40K on a Hiload 16/60 Superdex column (lane 7 to 11). Fractions 9 and 10 were pooled and further used in the *in vitro *killing assay.

As previously reported (Resch et al., 2011), Cpl-1^C45S;D194C ^generated the expected 37 kDa band plus a 74 kDa band on non-reducing SDS-PAGE (Figure 1, lane 2). The 74 kDa band vanished upon reduction with 10 mM DTT (Figure 1, lane 3) and therefore corresponded to a dimer. Indeed, dimerization was likely due to cysteine cross-bridging, thus indirectly indicating that the de novo introduced cysteines were properly exposed. A similar migration pattern was observed with all mutants in this study (data not shown). The seven fully active mutants (Resch et al., 2011) were further PEGylated. Figure 1 depicts a representative PEGylation experiment with PEG 40K. As determined by ImageJ (Abramoff et al., 2004), a small fraction of enzyme (3-12%, depending on the mutant), was not PEGylated (Figure 1, lane 5 for Cpl-1^C45S;D194C^). After gel filtration, fractions containing highly pure PEGylated conjugates were recovered (Figure 1, lane 9 and 10 for Cpl-1^C45S;D194C^) and pooled. The seven PEGylated conjugates lost 100% of their activity in the *in vitro *killing assay (data not shown), suggesting that the bulky effect of the PEG 40K molecule drastically interfered with enzyme function.

**Figure 2:**
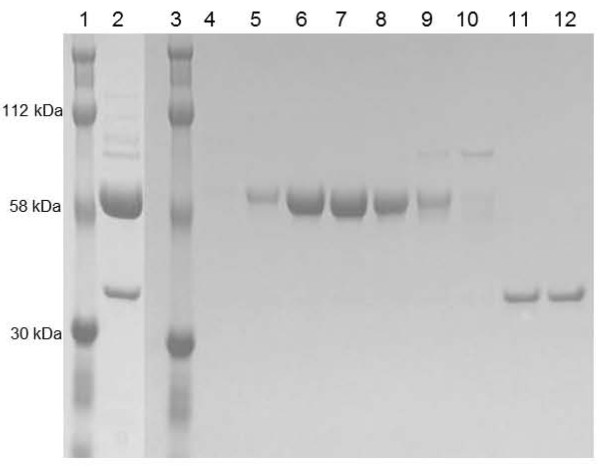
**Non-reducing SDS-PAGE of Cpl-1^C45S;D194C ^PEGylated with PEG 10K**. Protein ladder (lanes 1 and 3); Cpl-1^C45S;D194C ^PEGylated with PEG 10K and purified on a DEAE-sepharose column (lane 2); further purification of Cpl-1^C45S;D194C ^PEGylated with PEG 10K on a Hiload 16/60 Superdex column (lane 4 to 12). Fractions 6, 7, and 8 were pooled and further used in the *in vitro *killing assay. Residual non-PEGylated Cpl-1^C45S;D194C ^is shown (lane 11 and 12).

We reasoned that smaller adducts would be less detrimental to the enzyme, and therefore repeated the experiments with PEG 10K. Figure 2 depicts a representative PEGylation experiment with PEG 10K. This PEGylation reaction was also incomplete with 15-20% of residual non-PEGylated enzyme remaining in the mixture (Figure 2, lane 2 for Cpl-1^C45S;D194C^). Following gel filtration, fractions containing highly pure PEG 10K conjugates (Figure 2, lane 6, 7 and 8 for Cpl-1^C45S;D194C^) were separated from fractions containing non-PEGylated enzymes (Figure 2, lane 11 and 12 for Cpl-1^C45S;D194C^) and pooled. As for PEG 40K conjugates, none of the PEG 10K conjugates retained significant *in vitro *anti-microbial activity when tested in the *in vitro *killing assay (data not shown). The reduced electrophoretic migration of the PEG conjugates (ca.120 kDa instead of 77 kDa and ca. 60 kDa instead of 47 kDa for PEG 40K and PEG 10K conjugates; Figure 1, lane 5 and Figure 2, lane 2, respectively) might be attributed to steric hindrance of the PEG molecule.

## Discussion

While introducing cysteines at several sites on Cpl-1 did not alter its bactericidal activity, PEGylation on these residues totally abrogated it. This might be related to the complex structure and mode of action of the enzyme, which makes it susceptible to bulky adducts. Cpl-1 has a C-terminal domain that mediates binding to choline in the cell wall for adequate positioning of the N-terminal catalytic domain to cleave its substrate (Diaz et al., 1990; Perez-Dorado et al., 2007). Optimal positioning may also depend on enzyme C-terminus dimerization, as described for the pneumococcal autolysin LytA (Romero et al., 2007).

Susceptibility to PEG-related hindrance is supported by the fact that PEGylation on the hinge region (C194) inhibited activity, in spite of the fact that this region is independent of both the binding and active domains. Adding a bulky adduct to this location is thought to affect flexibility of the hinge and interfere with optimal orientation of the enzyme into the wall.

The present results do not preclude that PEGylation at other sites or with different types of PEG could possibly extend Cpl-1 half-life with less detrimental effect on its bactericidal activity. However, we believe that this work highlights the fact that cysteine-specific PEGylation could be unsuitable for a large set of enzybiotics with a similar architecture.

## Competing interests

The authors declare that they have no competing interests.
